# Identification of *ANKDD1B* variants in an ankylosing spondylitis pedigree and a sporadic patient

**DOI:** 10.1186/s12881-018-0622-9

**Published:** 2018-07-05

**Authors:** Zhiping Tan, Hui Zeng, Zhaofa Xu, Qi Tian, Xiaoyang Gao, Chuanman Zhou, Yu Zheng, Jian Wang, Guanghui Ling, Bing Wang, Yifeng Yang, Long Ma

**Affiliations:** 1 Clinical Center for Gene Diagnosis and Therapy, the Second Xiangya Hospital of Central South University, Changsha, 410011 China; 2 Department of Cardiovascular Surgery, the Second Xiangya Hospital of Central South University, Changsha, 410011 China; 3 Center for Medical Genetics, School of Life Sciences, Central South University, Changsha, 410081 China; 4 Department of Rheumatology, the Second Xiangya Hospital of Cenral South University, Changsha, China; 5 Department of Spine Surgery, the Second Xiangya Hospital Central South University, Changsha, 410011 Hunan China

**Keywords:** Ankylosing spondylitis, Pedigree, *ANKDD1B*, Ankyrin repeat, HLA-B*27

## Abstract

**Background:**

Ankylosing spondylitis (AS) is a debilitating autoimmune disease affecting tens of millions of people in the world. The genetics of AS is unclear. Analysis of rare AS pedigrees might facilitate our understanding of AS pathogenesis.

**Methods:**

We used genome-wide linkage analysis and whole-exome sequencing in combination with variant co-segregation verification and haplotype analysis to study an AS pedigree and a sporadic AS patient.

**Results:**

We identified a missense variant in the ankyrin repeat and death domain containing 1B gene *ANKDD1B* from a Han Chinese pedigree with dominantly inherited AS. This variant (p.L87V) co-segregates with all male patients of the pedigree. In females, the penetrance of the symptoms is incomplete with one identified patient out of 5 carriers, consistent with the reduced frequency of AS in females of the general population. We further identified a distinct missense variant affecting a conserved amino acid (p.R102L) of ANKDD1B in a male from 30 sporadic early onset AS patients. Both variants are absent in 500 normal controls. We determined the haplotypes of four major known AS risk loci, including HLA-B*27, *2p15*, *ERAP1* and *IL23R*, and found that only HLA-B*27 is strongly associated with patients in our cohort.

**Conclusions:**

Together these results suggest that *ANKDD1B* variants might be associated with AS and genetic analyses of more AS patients are warranted to verify this association.

**Electronic supplementary material:**

The online version of this article (10.1186/s12881-018-0622-9) contains supplementary material, which is available to authorized users.

## Background

Ankylosing spondylitis (AS) is a severe, debilitating and incurable autoimmune disease affecting multi millions of people in the world [1]. AS is the major subtype of spondyloarthritis, a spectrum of inter-related rheumatic diseases that also includes reactive arthritis (ReA), psoriatic arthritis (PsA), juvenile spondyloarthritis (JSpA), enteropathic arthritis (spondylitis/arthritis associated with inflammatory bowel disease), and undifferentiated spondyloarthritis (USpA) (https://www.spondylitis.org) [2, 3].

The symptoms of AS often appear gradually and progress from stiffness and chronic dull pain in the lower back to severe pain felt from the sacroiliac joint. In severe cases, AS can cause a complete fusion (ankylosis) of the spine. Many AS patients suffer severe loss of mobility and as a consequence lose working capabilities.

The average AS prevalence varies among different populations, with 0.24% in Europe, 0.17% in Asia, 0.3% in North America, 0.1% in Latin America and 0.7% in Africa. Based on these ratios, it is estimated that the number of AS cases in Europe and Asia alone could reach 1.30–1.56 million and 4.63–4.98 million, respectively [1].

AS is highly inheritable. The recurrence risks in monozygous twins and first-degree relatives are 63 and 8.2%, respectively [4, 5]. Most AS is presented in sporadic cases, probably reflecting the oligogenic nature of this disease. The human leukocyte antigen B27 (HLA-B*27) genotype was found strongly associated with AS [6–8]. Specifically HLA-B*27 carriers could have a 20-fold increase in the risk of developing spondylarthropathy-related diseases [9], which is exemplified by the fact that most AS patients are HLA-B*27 positive in the general population. However the presence of HLA-B*27 genotype is not sufficient for AS pathogenesis, as only 1–5% HLA-B*27 carriers eventually develop AS [8, 10, 11].

Recently large-scale genome-wide association studies on patients with European ancestry and of the Han Chinese have identified at least 31 non*-HLA-B* genetic loci associated with AS [11–17]. Among these loci, *IL23R*, *2p15*, *ERAP1* exhibit the most significant association [12, 15–17]. Nevertheless these loci, together with HLA-B*27, could explain only 24.4% of the heritability of AS [15]. Therefore the major genetic causes of AS remain to be identified. Ideally, AS cases from consanguinity inheritance or large pedigrees could provide a simpler inheritance pattern compared to sporadic cases, which might facilitate the identification of more elusive risk genes of AS.

To further understand the genetics of AS, we employed a combination of genome-wide linkage analysis and next-generation sequencing (NGS) in a three-generation Han Chinese pedigree with five AS patients. The analysis revealed a missense variant affecting a conserved amino acid in the novel gene *ANKDD1B* that segregates with the disease. We further identified a distinct *ANKDD1B* missense variant in a male by surveying a group of sporadic AS patients using exome sequencing. These findings suggest that *ANKDD1B* variants might be related with the pathogenesis of AS.

## Methods

### Patients and subjects

**Fig. 1:**
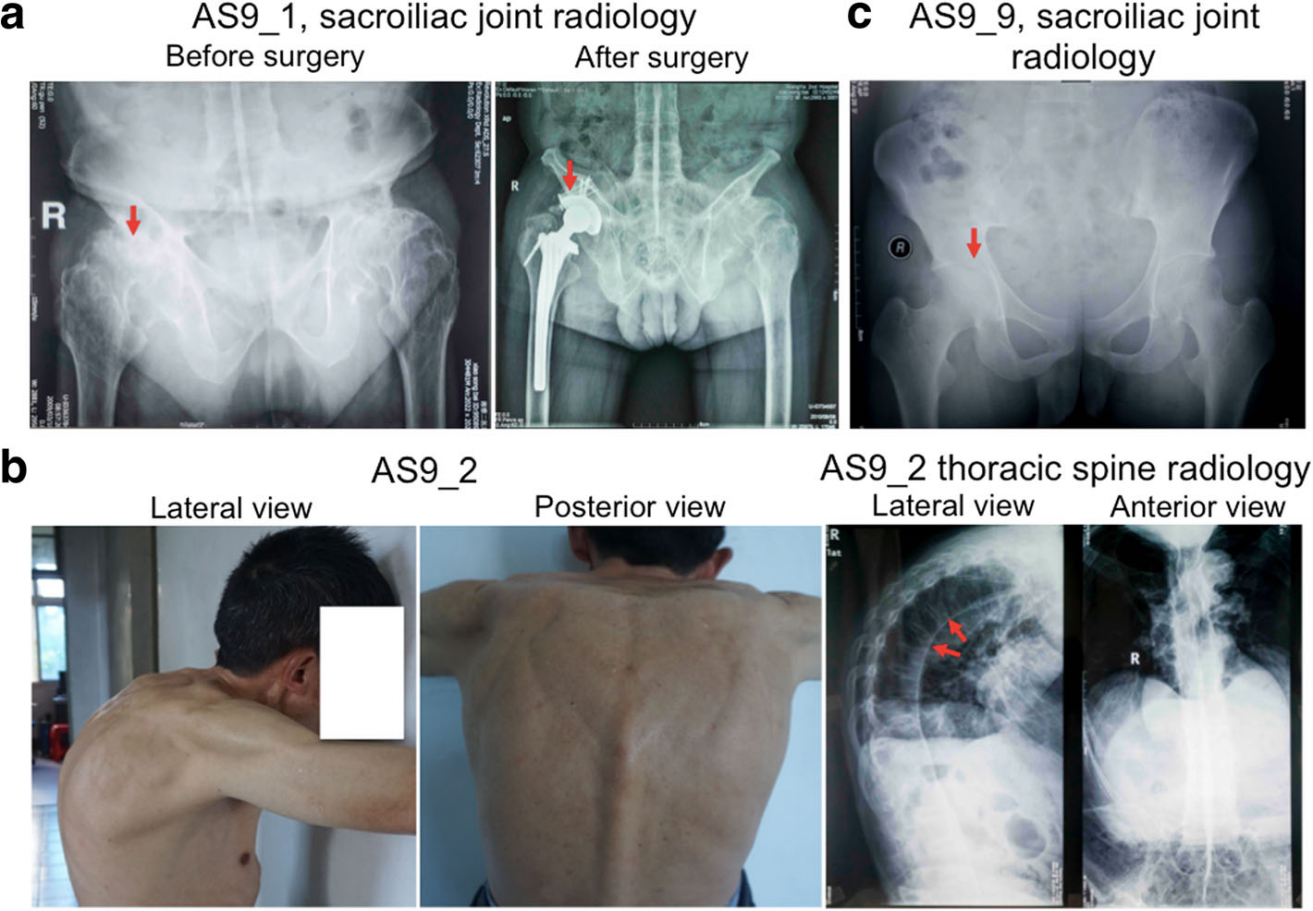
Medical images of AS9 patients. **a** X-ray pictures of the sacroiliac joints of the proband, AS9_1, before (left) and after (right) joint replacement surgery. Arrows indicate erosion of the right joint before the surgery (left) and the artificial joint after the surgery (right). **b** Medical images of patient AS9_2, showing the deformation of the thoracic spine due to ankylosis (left). X-ray pictures showing the “bamboo”-like spines of AS9_2 (right). Arrows point to the sites of fused vertebrae. **c** Sacroiliitis of patient AS9_9 detected by X-ray photography

**Fig. 2:**
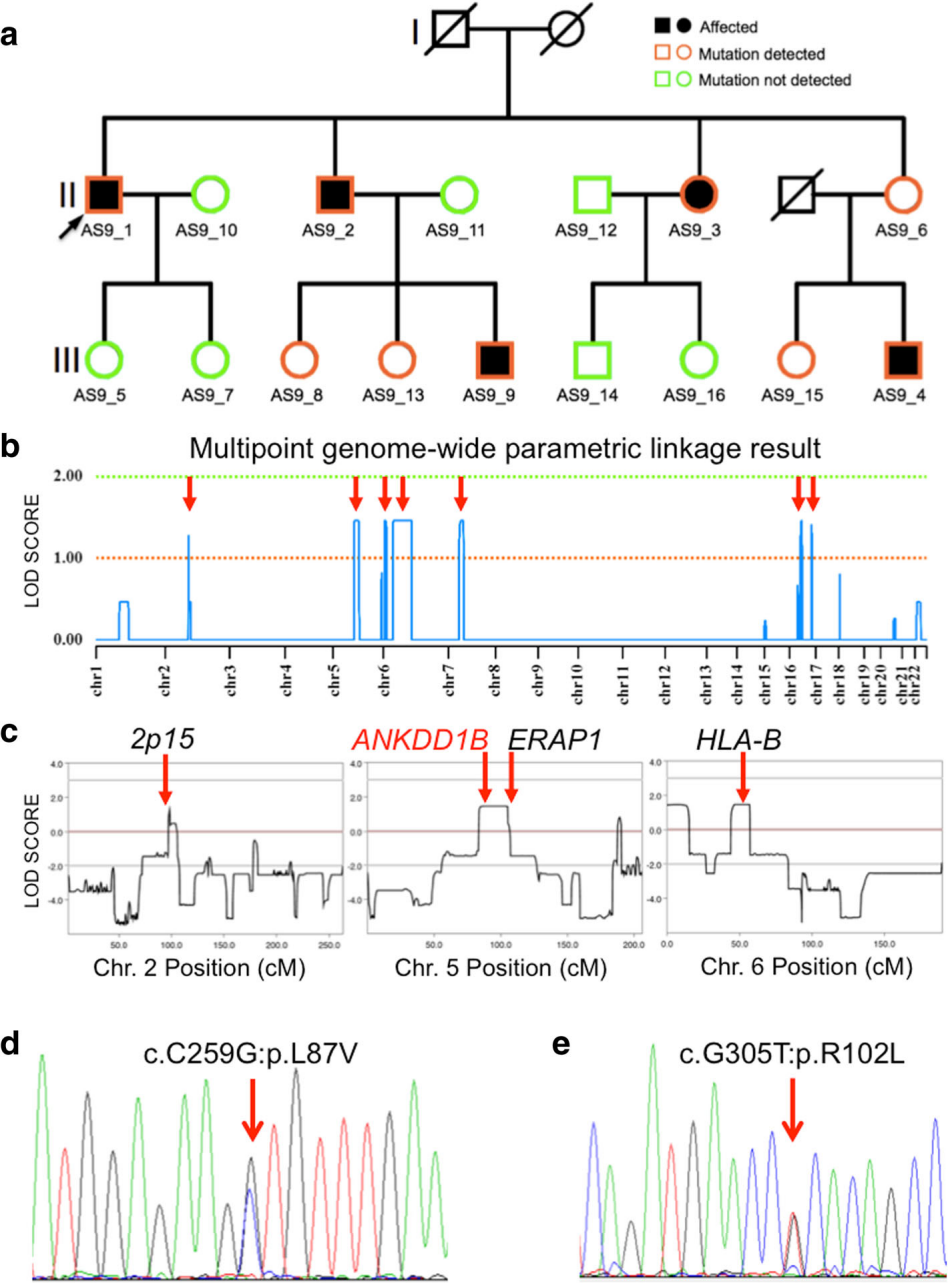
Whole-genome linkage analysis and exome sequencing identified *ANKDD1B* to be associated with AS. **a** The AS9 pedigree. Generations, non-carriers, non-symptomatic carriers and patients are indicated. Arrow points to the proband. **b** Whole-genome linkage analysis identified seven regions (arrows) on Chr. 2, Chr. 5, Chr. 6, Chr. 7 and Chr. 16 to be significantly linked to disease transmission in the AS9 pedigree. **c** A delineation of the *ANKDD1B* locus and the major known AS risk loci in relation to the linkage regions. **d**, **e** The L87V variant and the R102L variant in *ANKDD1B* as shown in Sanger sequencing chromatograms

The study protocol was approved by the Review Board of the Second Xiangya Hospital of the Central South University in China with informed consent from each study participant. The proband (AS9_1) (Fig. 1) was diagnosed with ankylosing spondylitis in 2009 at the Department of Rheumatology of the Second Xiangya Hospital. A follow up of the proband identified a 16-member, three-generation AS9 pedigree (Fig. 2a). The disease history of the five AS9 patients and the sporadic patient sAS_P1 is shown in Additional file 1: Table S1. Medical images of two other patients are also shown in Fig. 1.

### DNA extraction

Genomic DNA was extracted from peripheral blood of the family members using a DNeasy Blood & Tissue Kit (Qiagen, Valencia, CA) on the QIAcube automated DNA extraction robot (Qiagen, Hilden, Germany).

### Genome-wide linkage analysis

Genomic DNA samples of the AS9 family members were adjusted to a final concentration of 50 ng/μl. The Infinium OmniZhongHua-8 v1.3 Beadchip (Illumina Inc., San Diego, USA) and the Illumina BeadScan genotyping system (Beadstation Scanner) were employed to obtain the signal intensities of SNP probes. The Infinium OmniZhongHua-8 v1.3 Beadchip contains 887,270 SNPs that cover common, intermediate and rare variants specific to Chinese populations. The GenomeStudio V1.0 Genotyping Module was used to analyze the genotypes (human genome build 37/Hg19) and evaluate the experimental quality. The call rates of the samples were greater than 99.5%. After no call SNPs, SNPs with minor allele frequency < 0.05, and mismatched SNPs between parents and children were excluded, we chose one SNP per 0.5 cM (7079 SNPs across the genome with an average gap of 379 kb) for multi-point parametric linkage analysis using the merlin (v1.1.2) software based on an autosomal dominant inheritance disease model. Specifically we excluded all females of the third generation from the linkage analysis, since females have a penetrance much lower than males [2]. We treated AS9_6 as patient because she has a symptomatic son.

### Whole-exome sequencing

Four members of the AS9 family (AS9_1, AS9_2, AS9_3 and AS9_4) were subjected to whole-exome sequencing analysis. Each individual paired-end Agilent SureSelect library was prepared according to manufacturer’s instructions (Agilent) from 1.0 μg genomic DNA sheared with a Covaris S220 sonicator (Covaris, Inc., Woburn, USA). Exome capture was performed with Agilent’s SureSelect Human All Exon kit V5, which resulted in ~ 50 Mb DNA sequences of 334,378 exons from 20,965 genes being captured. Paired-end sequencing (150-bp reads) was carried on HiSeq 4000 platform (Illumina). After demultiplexing, paired-end sequences were mapped to the human genome (UCSC hg19) using Burrows-Wheeler Aligner (BWA) [18]. The mean coverage of the target regions obtained for the four samples was 99.9, 99.9, 99.8 and 99.9%, with average sequencing depth of 90.86X, 83.27X, 76.24X and 83.87X for AS9_1, AS9_2, AS9_3 and AS9_4, respectively (Additional file 2: Table S2). Downstream processing was performed using the Genome Analysis Toolkit (GATK), varscan2 and Picard, and variant calls were made with the GATK HaplotypeCaller. Variant annotation was based on Ensembl release 82, and filtering was performed with ANNOVAR Documentation.

127,303, 122,644, 115,062 and 126,720 confidence variants in AS9_1, AS9_2, AS9_3 and AS9_4, respectively, were identified from the exome sequencing (Additional file 3: Table S3). Non-synonymous SNPs or frameshift-causing INDELs with an alternative allele frequency > 0.005 in the NHLBI Exome Sequencing Project Exome Variant Server (ESP6500), dbSNP (build 138) (http://www.ncbi.nlm.nih.gov/projects/SNP/index.html), the 1000 Genomes Browser (released May 2012) (http://www.1000genomes.org/) or the ExAC Browser (http://exac.broadinstitute.org) were excluded prior to analysis. The called SNVs and INDELs were filtered and those predicted by HapMap Genome Browser (release #28) (http://hapmap.ncbi.nlm.nih.gov/), SIFT (http://sift.jcvi.org/), Polyphen2 (http://genetics.bwh.harvard.edu/pph2/) and MutationTaster (http://www.mutationtaster.org/) to be non-deleterious were excluded. By this approach, six candidate variants distributed in five genes were identified in all four patients (Additional file 3: Table S3 and Additional file 4: Table S4), which include *GBP5* (containing two distinct variants), *ANKDD1B*, *TMEM30B*, *CACNA1H* and *RFPL3.*

### Variant validation and co-segregation analysis

Sanger sequencing was used to validate the candidate variants found in exome sequencing. Segregation analyses were performed on the family members. The two *ANKDD1B* variants were also examined in the exome sequences of 500 healthy adults of both sexes and different ages, who were recruited by the genome sequencing company Novogene (Beijing) and used as an internal control for genetic variants potentially specific for the Han Chinese. Primer pairs used to amplify fragments encompassing individual variants were designed using an online tool (PrimerQuest, IDT) (http://www.idtdna.com/Primerquest/Home/Index) and the primer sequences for *ANKDD1B* are listed in Additional file 5: Table S5.

### Haplotype verification

The genomic sequence for each SNP was obtained from dbSNP database (https://www.ncbi.nlm.nih.gov/projects/SNP/). The genomic DNAs from patients were amplified by PCR using primers listed in Additional file 6: Table S6 and the sequences of the amplified fragments were determined.

## Results

### The AS9 ankylosing spondylitis pedigree

Using the modified New York criteria for ankylosing spondylitis [19], a diagnosis of AS was made on three individuals (AS9_1, AS9_2, AS9_9) of a three-generation pedigree of the Han Chinese (Fig. 1, Fig. 2a and Additional file 1**:** Table S1). The family history suggests an autosomal dominant pattern of inheritance, which predominantly affects male members (Fig. 2a). The shared symptoms include: the onset of chronic lower back pain before the age of 20 years, symptomatic sacroiliitis (persistent pain and stiffness lasting > 3 months) and improvement on exercise and worsening with rest. In the proband AS9_1, the symptom was limited to sacroiliitis on both sides, which was verified by radiology (Fig.1a, left). Joint replacement surgery was performed on the right sacroiliac joint of the proband in 2010 (Fig. 1a, right). The proband described that his deceased father also had similar symptoms. For patient AS9_2, the progression of the disease has led to severe deformation of the spine (Fig. 1b, left) and the formation of the “bamboo”-like vertebral joints due to ankylosis (Fig. 1b, right). Patient AS9_9 is the son of patient AS9_2 and began to show mild signs of sacroiliitis before the age of 20 years, which was confirmed by radiology (Fig. 1c).

The other two patients (AS9_3 and AS9_4) were diagnosed based on symptoms that include teenage onset of chronic back pain and symptomatic sacroiliitis (Additional file 1: Table S1). However these symptoms were not verified by radiology due to personal reasons (Additional file 1: Table S1). AS9_3 was not tested for HLA-B*27 positivity as well. We later genotyped rs13202464, a SNP associated with the *HLA-B* locus in the Han Chinese [16] and rs116488202, another verified SNP strongly associated with both Europeans and the Han Chinese [15] (Table 1). We found that AS9_3 shares identical haplotypes at these loci with other HLA-B*27-positive patients of the family, suggesting that AS9_3 is likely HLA-B*27-positive as well.

**Table 1:** Haplotype analysis of major AS risk loci on the study subjects

ID	Phenotype	Sex	ANKDD1B	HLA-B27	HLA-B27	2p15	ERAP1	IL23R
				rs13202464	rs116488202	rs6759298	rs30187	rs11209026
AS9_1	AS	M	L87 V/+	G/A	C/C	C/G	C/C	G/G
AS9_2	AS	M	L87 V/+	G/A	C/C	C/G	C/C	G/G
AS9_4	AS	M	L87 V/+	G/A	C/C	G/G	T/C	G/G
AS9_9	AS	M	L87 V/+	G/A	C/C	C/G	T/C	G/G
sAS_P1	AS	M	R102L/+	G/A	C/C	G/G	C/C	G/G
AS9_3	AS	F	L87 V/+	G/A	C/C	G/G	T/C	G/G
AS9_6	N	F	L87 V/+	G/A	C/C	G/G	C/C	G/G
AS9_8	N	F	L87 V/+	G/A	C/C	C/G	T/C	G/G
AS9_15	N	F	L87 V/+	G/A	C/C	G/G	T/C	G/G
AS9_13	N	F	L87 V/+	A/A	C/C	C/G	T/C	G/G
AS9_5	N	F	WT	A/A	C/C	G/G	C/C	G/G
AS9_7	N	F	WT	A/A	C/C	G/G	T/C	G/G
AS9_12	N	M	WT	A/A	C/C	C/G	T/C	G/G
AS9_10	N	F	WT	A/A	C/C	C/G	T/C	G/G
AS9_11	N	F	WT	A/A	C/C	C/G	T/C	G/G
AS9_14	N	M	WT	A/A	C/C	G/G	T/C	G/G
AS9_16	N	F	WT	A/A	C/C	C/G	T/C	G/G

### Genome-wide linkage analysis identified seven risk chromosomal regions in the AS9 pedigree

AS is an oligogenic disease [11] and most AS patients are identified as sporadic cases. The heritability of AS in the AS9 pedigree might provide a rare opportunity for identifying new AS-associated genetic variants.

To identify the potential genetic variants, we performed genome-wide linkage analysis on 10 AS9 family members (six third-generation females were excluded due to the incomplete penetrance of AS in females) based on an autosomal dominant inheritance model (see Methods). Multi-point parametric linkage analysis identified seven regions on five chromosomes with maximum multi-point parametric LOD (logarithm of odds) scores between 1.275 and 1.4608 (Fig. 2b and Table 2). We also identified seven other regions distributed on seven chromosomes, with the maximal LOD scores between 0.2322 and 0.8175 (Fig. 2b).

**Table 2:** List of linkage regions with MAX-LOD scores above 1.0

Chr.	Left SNP	Right SNP	MAX-LOD
2	rs17007729	rs17018719	1.275
5	rs3112483	rs13161885	1.4608
6	rs7753332	rs1590328	1.4571
6	rs4710988	rs259686	1.4608
7	rs739749	rs13222756	1.4607
16	rs16947530	rs7184310	1.4598
16	rs17673125	rs16961072	1.4069

### Whole-exome sequencing identified six candidate variants in the AS9 pedigree

The linkage analysis only provided information concerning the genomic locations of potential genetic variants associated with the AS9 pedigree. To narrow down the genetic variants, we performed exome sequencing on four patients (AS9_1, AS9_2, AS9_3 and AS9_4). In total we obtained on average 4.6 Gbp, 4.2 Gbp, 3.8 Gbp and 4.2 Gbp sequences that covered more than 99% of the exonic regions for AS9_1, AS9_2, AS9_3 and AS9_4, respectively (Additional file 2: Table S2). The sequencing depth for each patient was from 76X for AS9_3 to 90X for AS9_1.

From the exome sequencing (see Methods), we identified six candidate variants distributed in five genes that are shared by all four patients (Additional file 3: Table S3 and Additional file 4: Table S4). These genes include *GBP5* (containing two different variants), *ANKDD1B*, *TMEM30B*, *CACNA1H* and *RFPL3.*

We examined co-segregation of these variants with individuals of the AS9 pedigree and found that the *CACNA1H* variant was absent in the AS9_9 patient, suggesting that *CACNA1H* is probably not a risk locus. MutationTaster (www.mutationtaster.org) prediction indicates that the variants in *GBP5* and *RFPL3* are likely non-disease causing polymorphisms. The variant in *TMEM30B* was reported in the ExAC database (http://exac.broadinsitutute.org) at a frequency of 0.002 and is predicted by the SIFT software (www.sift.jcvi.org) to be a highly tolerated change. We currently could not conclude whether this variant confers any risk for AS. Together these analyses suggest that the genetic variant in *ANKDD1B* is worthy of further consideration.

### *ANKDD1B* variants might be associated with ankylosing spondylitis

We next compared the genomic positions of the five candidate genes (Additional file 4: Table S4) with the whole-genome linkage results (Fig. 2b and c). Interestingly only *ANKDD1B* is contained within one of the seven linkage regions with MAX_LOD score above 1.275 (Table 2), which is between rs3112483 and rs13161885 on Chr. 5 (Fig. 2c and Table 2). This result is consistent with the bioinformatics analysis described above and suggests that *ANKDD1B* is potentially associated with the AS9 pedigree.

We examined the coding exons of *ANKDD1B* in all 16 members of the AS9 family by Sanger sequencing. The L87V variant (Fig. 2d) was verified in the four patients analyzed by exome sequencing and also patient AS9_9 (Fig. 2a), who was not included in our initial exome analysis. The variant was found in AS9_6, who is non-symptomatic but has a symptomatic son. We further detected the L87V variant in three non-symptomatic members of the family (Fig. 2a). Two are daughters of patient AS9_2 and one is the daughter of the non-symptomatic AS9_6. This variant was not detected in seven other non-symptomatic family members (Fig. 2a and Table 1).

*ANKDD1B* encodes a novel conserved protein with multiple ankyrin repeats and a death domain (Additional file 7: Figure S1). The variant found in the AS9 pedigree changes a conserved leucine to valine at position 87 within the first ankyrin repeat and is predicted to cause a partially deleterious effect on the protein function (www.predictprotein.org).

To further examine the association of *ANKDD1B* with AS, we surveyed the coding exons and splice sites of *ANKDD1B* in 30 sporadic AS patients previously analyzed by whole-exome sequencing (Z. Tan and H. Zeng, unpublished observations). From a 21-year old male patient (sAS_P1, Additional file 1: Table S1), we identified a distinct missense variant that changes a conserved arginine to leucine at position 102 (exon3:c.G305T:p.R102L) within the second ankyrin repeat (Fig. 2e, Table 1 and Additional file 7: Figure S1). This variant is predicted to cause strongly deleterious effect on the protein function (www.predictprotein.org).

The two *ANKDD1B* variants were not found in 500 internal (local) control exomes sequenced. The L87 V variant identified in the pedigree was not identified in NHLBI Exome Sequencing Project Exome Variant Server (ESP6500), the 1000 Genomes Browser as well as the ExAC database. The variant identified in the sporadic male patient (sAS_P1) (c.G305 T:p.R102L) has a SNP number (rs191940699) and is presented in the dbSNP database (www.ncbi.nlm.nih.gov) with a global MAF of 0.0016/8. In addition this SNP is presented in the ExAc database (http://exac.broadinsitutute.org) as a c.G305A:p.R102H variant with an allele frequency of 7.477e-05 (1 out of 13,374 alleles from 6687 individuals in total). In the South Asian population in which this SNP was identified, its allele frequency is 0.0001422 (1 out of 7030 alleles from 3515 individuals). The low values of these frequencies imply a selection against these variants in the general population.

### Haplotype analysis of the *ANKDD1B* variant carriers

Besides the linkage region on Chr. 5 that contains *ANKDD1B*, we also identified six other regions (Table 2 and Fig. 2b) with MAX-LOD scores above 1.275. Interestingly, one region on Chr. 6 contains the *HLA-B* locus (Fig. 2c), prompting us to examine the haplotype of HLA-B*27 in our cohort. We found that the SNP rs13202464 previously reported to tag HLA-B*27 in Asian populations [16] also predominantly tags the patients (6/6 in patients vs 3/11 in non-AS individuals) (Table 1). However the SNP rs116488202 reported to tag HLA-B*27 more significantly in both Asian and European populations [15] is presented identically among the patients and normal individuals (Table 1), with the risk minor allele (T) absent in all individuals.

The linkage region on Chr. 2 does not contain the major AS risk locus *2p15* but is at a close distance, with 0.97 Mb between the left outline of this region and the SNP rs6759298 reported to tag *2p15* [15]. We genotyped rs6759298 and found that the risk allele of this SNP does not exhibit an apparent association with AS patients (3/6) vs non-AS individuals (6/11) (Table 1).

The *ERAP1* locus is also significantly associated with AS in the general population [12]. The *ERAP1*-tagging SNP rs30187 [15] is 0.3 Mb apart from the right outline of the linkage region on Chr. 5 (Fig. 2c). 3/6 patients carry the risk allele of rs30187 while 9/11 non-AS individuals are also positive (Table 1), suggesting that the genotype of *ERAP1* might not be associated with AS in our cohort.

No linkage region on Chr. 1 with a MAX-LOD score above 1.0 was identified. However since the AS risk locus *IL23R* is located on Chr. 1, we also genotyped the SNP rs11209026 that tags *IL23R* [15]. We found that all individuals in our cohort are homozygous for the risk allele (Table 1). Therefore we could not evaluate the contribution of the *IL23R* locus to the pathogenesis of our patients.

We compared the other four linkage regions (Table 2, in blue) with all the AS risk loci identified to date [11] and found that none are located within these regions. We postulate that these linkage regions might contain new risk loci for AS or are merely associated with the AS9 family by coincidence.

## Discussion

In this study, we combined genome-wide linkage analysis and exome sequencing to identify the *ANKDD1B* gene as a potential locus related ankylosing spondylitis in a Chinese AS pedigree.

AS is an oligogenic disease with over 75% of the heritability unexplained [11]. Of the 32 identified loci associated with AS [11, 15], HLA-B*27 is the most significant risk factor, followed by *IL23R*, *2p15* and *ERAP1* [15]. Among the seven linkage regions we identified in AS9 pedigree (Fig. 2b), one contains the *HLA-B* locus and two others are closely linked to the *2p15* and *ERAP1* loci, respectively (Fig. 2c). This correlation does not appear to be a random coincidence. Instead, it suggests that these linkage regions more or less correspond to the risk loci of AS and could be useful for distinguishing other genetic variants related to AS, e.g., those variants derived from exome sequencing. Indeed, among the six candidate variants that we identified from exome sequencing of the AS9 pedigree, only *ANKDD1B* is contained within a linkage region on Chr. 5 (Fig. 2c). Furthermore, a different *ANKDD1B* variant (R102L) was identified in a sporadic AS patient, emphasizing the potential correlation between *ANKDD1B* variants and AS in the Chinese patients.

The genetic location of *ANKDD1B* is *5q13.3* (www.ensembl.org), which is 0.1 cM away from *5q14.3,* a previously identified risk locus in Asian populations [16]. The significance of *5q14.3* locus was not replicated in the study of European populations [15]. Therefore it is unclear whether *5q14.3* represents an Asian-specific locus or the association of this locus with AS [16] was in fact caused by its closeness with *ANKDD1B*. The linkage region on Chr. 5 identified in this study is also close to but does not contain the risk locus *ERAP1* (Fig. 2c). Whether the haplotype of *ERAP1* affects the identification of this linkage region is unknown. The linkage region that we identified on Chr. 2 is close to another major AS risk locus, *2p15* [15] (Fig. 2c). Currently we could not determine whether the identification of this linkage region is caused by its closeness to *2p15* or it represents a different risk locus for AS. The other linkage regions (Table 2, in blue) do not contain any of the AS risk loci identified so far [11].

*ANKDD1B* encodes a novel protein containing 10 tandem ankyrin repeats and a death domain and is conserved from zebrafish to human (Additional file 7: Figure S1). Ankyrin repeats are found in proteins involved in inflammatory response, transcription, cell-cycle regulation, cytoskeleton integrity development, cell-cell signaling and protein transport [20]. Death domains are usually regulators of inflammation, innate immune response and cell death through their interactions with TNF (tumor necrosis factor) receptors and Toll-like receptors [21–23].

A protein expression database (www.proteinatlas.org) indicates that the ANKDD1B protein is expressed in muscles, distinct cells of the lymph node and tonsil, a chronic myeloid leukemia cell line (K-562), a multiple myeloma cell line (LP-1) and an ovarian cystadenocarcinoma cell line (EFO-21). In addition the *ANKDD1B* transcript was detected in multiple human tissues including the lymph node and spleen (www.genecards.org). In mouse, the *ANKDD1B* transcript is expressed in higher levels in multiple B- and T-cell lines and in various parts of the nervous system (www.biogps.org). The site of expression and the domain structure of ANKDD1B suggest a function in the immune system.

The complex oligogenic nature of AS hinders the genetic analysis of AS pathogenesis. A previous analysis of AS recurrence in relation to genetic distances estimated that multiplicative interactions involving ~five genetic loci could partially explain the occurrence of AS in the general population [5]. In our pedigree and the sporadic male patient, the presence of the *ANKDD1B* variant and HLA-B*27 are the most significant factors predicting whether an individual develops AS (Table 1), while the roles of three other GWAS-derived significant loci (*IL23R*, *2p15*, *ERAP1*) could not be evaluated, probably due to the limited size of our cohort.

In the AS9 pedigree, all four male carriers are HLA-B*27 positive and developed AS (Fig. 2a and Table 1). Four of the five female carriers are HLA-B*27 positive, while only one developed AS (Fig. 2a and Table 1). This phenomenon is consistent with AS epidemiology in the general population, in which males are three times more frequently affected than females [2].

Besides the AS9 pedigree described in our study, three other Han Chinese AS pedigrees were also described recently [24], in which the *2q36.1-36.3* locus was found to be associated with disease transmission in addition to HLA-B*27. This locus has not been associated with AS in other association studies [11]. It is possible that a combination of genome-wide linkage analysis and genome sequencing would narrow down the potential AS-related genetic variants in these pedigrees.

## Conclusions

In short, we found that a novel missense variant of the *ANKDD1B* gene might be associated with patients in a rare AS pedigree of the Han Chinese. We also found a different *ANKDD1B* missense variant in a sporadic AS patient. *ANKDD1B* was identified with the combination of genome-wide linkage analysis and exome sequencing and verified to be co-segregated with the patients in a similar manner as the HLA-B*27 locus, implying that this gene might be related to AS pathogenesis and is worth to be considered for understanding the genetics of AS. Future studies, including verification of this association in more AS patients and analysis of animal models carrying *ANKDD1B* mutations, might provide novel insights into the pathogenesis of AS.

## Additional files

Additional file 1: **Table S1.** List of patients from the AS9 pedigree and sAS_P1. (PPTX 45 kb)

Additional file 2: **Table S2.** Exome sequencing quality metrics. (PPTX 46 kb)

Additional file 3: **Table S3.** NGS summary. (PPTX 54 kb)

Additional file 4: **Table S4.** List of candidate genes. (PPTX 46 kb)

Additional file 5: **Table S5.** *ANKDD1B* PCR and sequencing primers. (PPTX 40 kb)

Additional file 6: **Table S6.** PCR primers for haplotype genotyping. (PPTX 41 kb)

Additional file 7: **Figure S1.** ANKDD1B protein domains and sequence alignment. Upper panel: human ANKDD1B domain structure. AR: ankyrin repeat. Lower panel: ANKDD1B protein sequence alignment from zebrafish to human. The two variants identified in the AS9 pedigree and the sAS_P1 patient are indicated. (TIFF 6077 kb)
