# Supplementary material for: Identification of ANKDD1B variants in an ankylosing spondylitis pedigree and a sporadic patient
Source: BMC Med Genet. 2018 Jul 5;19:111. doi: 10.1186/s12881-018-0622-9 (PMC6034262; doi:10.1186/s12881-018-0622-9)
Supplement: Supplementary file 1 — Table S1. List of patients from the AS9 pedigree and sAS_P1. (PPTX 45 kb) [file 12881_2018_622_MOESM1_ESM.pptx]

## Slide 1
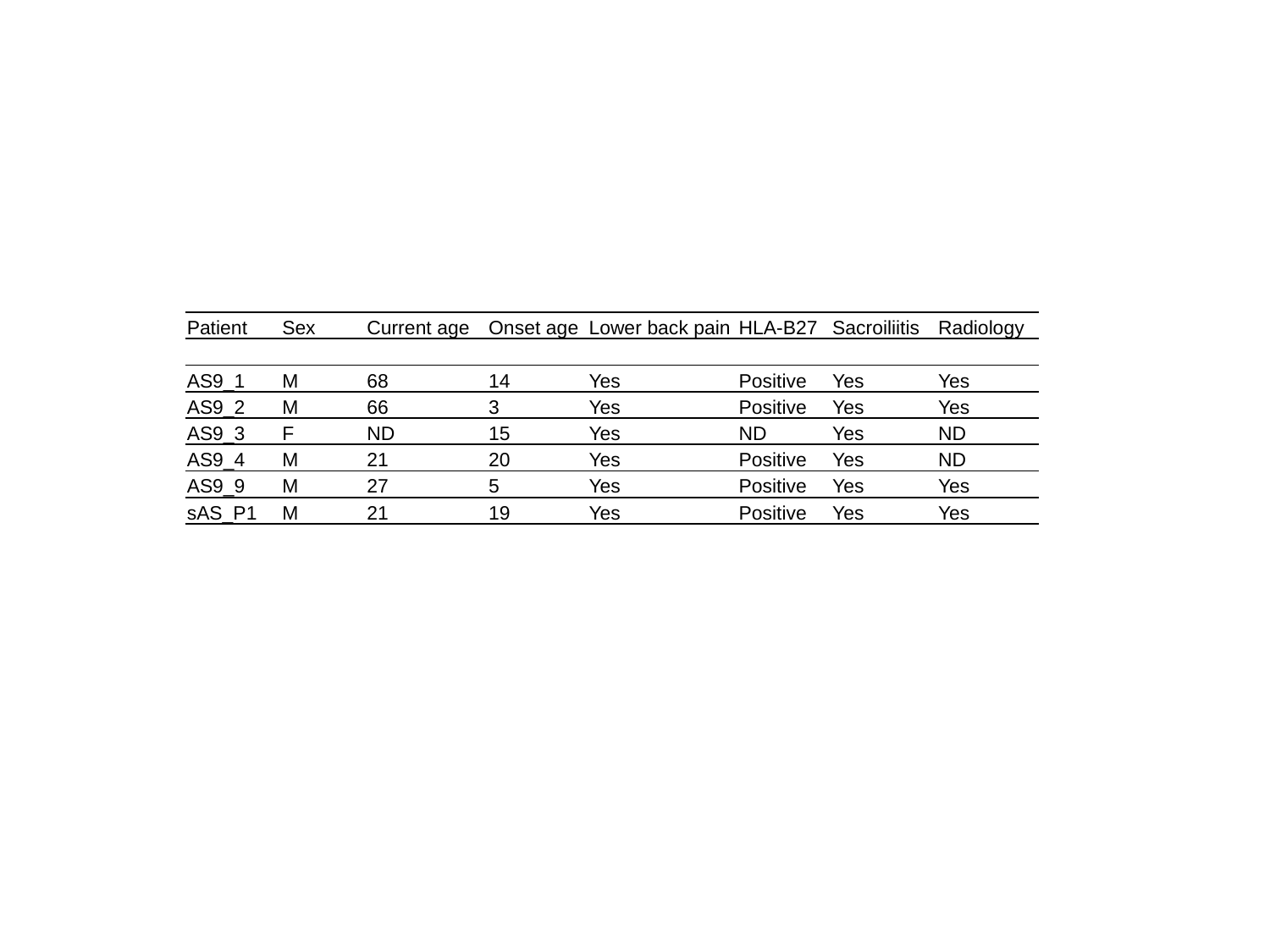

| | | | | | | | |
| --- | --- | --- | --- | --- | --- | --- | --- |
| Patient | Sex | Current age | Onset age | Lower back pain | HLA-B27 | Sacroiliitis | Radiology |
| | | | | | | | |
| AS9\_1 | M | 68 | 14 | Yes | Positive | Yes | Yes |
| AS9\_2 | M | 66 | 3 | Yes | Positive | Yes | Yes |
| AS9\_3 | F | ND | 15 | Yes | ND | Yes | ND |
| AS9\_4 | M | 21 | 20 | Yes | Positive | Yes | ND |
| AS9\_9 | M | 27 | 5 | Yes | Positive | Yes | Yes |
| sAS\_P1 | M | 21 | 19 | Yes | Positive | Yes | Yes |
