# Supplementary material for: Identification of ANKDD1B variants in an ankylosing spondylitis pedigree and a sporadic patient
Source: BMC Med Genet. 2018 Jul 5;19:111. doi: 10.1186/s12881-018-0622-9 (PMC6034262; doi:10.1186/s12881-018-0622-9)
Supplement: Supplementary file 2 — Table S2. Exome sequencing quality metrics. (PPTX 46 kb) [file 12881_2018_622_MOESM2_ESM.pptx]

## Slide 1
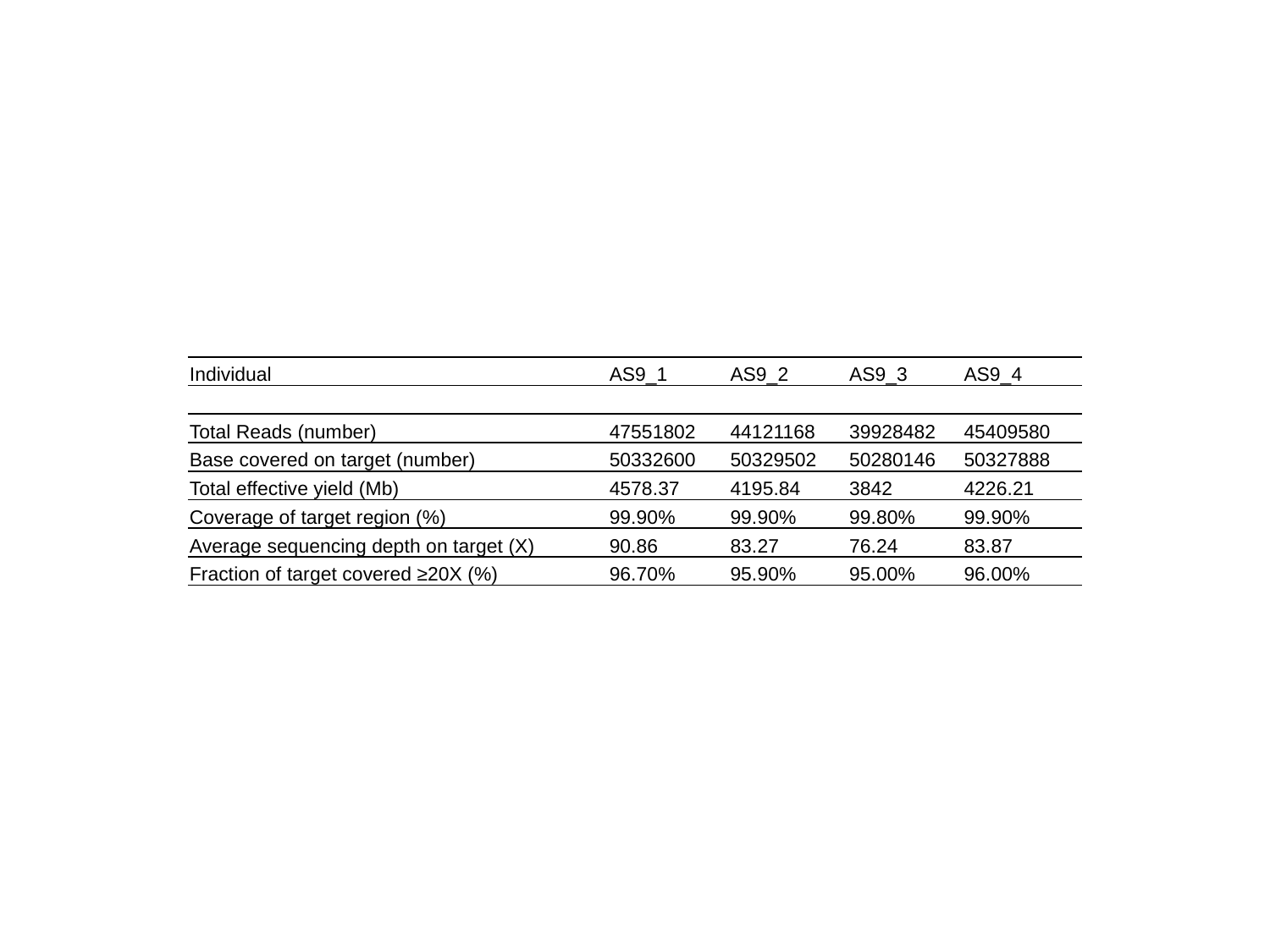

| | | | | |
| --- | --- | --- | --- | --- |
| Individual | AS9\_1 | AS9\_2 | AS9\_3 | AS9\_4 |
| | | | | |
| Total Reads (number) | 47551802 | 44121168 | 39928482 | 45409580 |
| Base covered on target (number) | 50332600 | 50329502 | 50280146 | 50327888 |
| Total effective yield (Mb) | 4578.37 | 4195.84 | 3842 | 4226.21 |
| Coverage of target region (%) | 99.90% | 99.90% | 99.80% | 99.90% |
| Average sequencing depth on target (X) | 90.86 | 83.27 | 76.24 | 83.87 |
| Fraction of target covered ≥20X (%) | 96.70% | 95.90% | 95.00% | 96.00% |
