# Supplementary material for: Identification of ANKDD1B variants in an ankylosing spondylitis pedigree and a sporadic patient
Source: BMC Med Genet. 2018 Jul 5;19:111. doi: 10.1186/s12881-018-0622-9 (PMC6034262; doi:10.1186/s12881-018-0622-9)
Supplement: Supplementary file 3 — Table S3. NGS summary. (PPTX 54 kb) [file 12881_2018_622_MOESM3_ESM.pptx]

## Slide 1
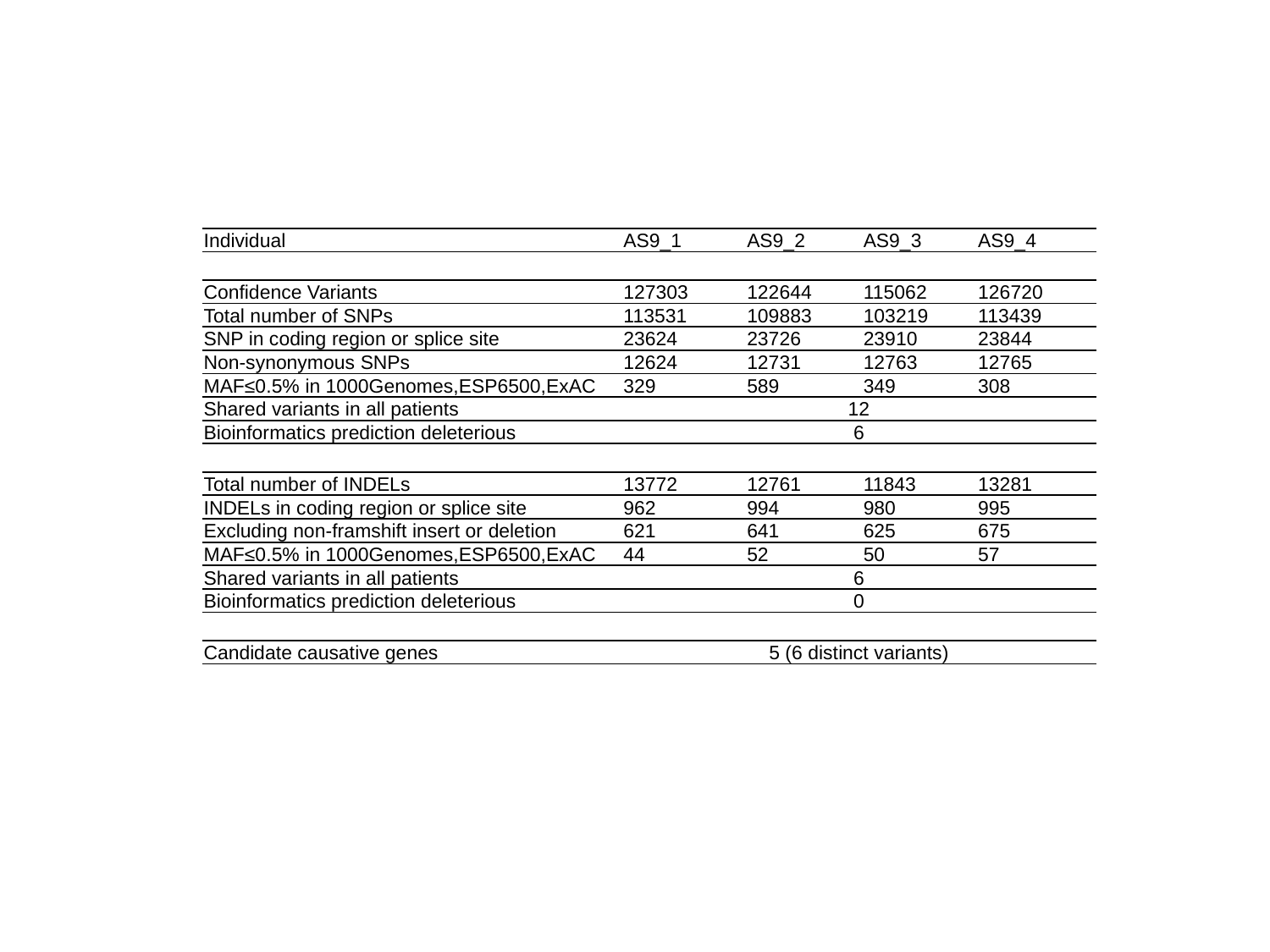

| | | | | |
| --- | --- | --- | --- | --- |
| Individual | AS9\_1 | AS9\_2 | AS9\_3 | AS9\_4 |
| | | | | |
| Confidence Variants | 127303 | 122644 | 115062 | 126720 |
| Total number of SNPs | 113531 | 109883 | 103219 | 113439 |
| SNP in coding region or splice site | 23624 | 23726 | 23910 | 23844 |
| Non-synonymous SNPs | 12624 | 12731 | 12763 | 12765 |
| MAF≤0.5% in 1000Genomes,ESP6500,ExAC | 329 | 589 | 349 | 308 |
| Shared variants in all patients | 12 | | | |
| Bioinformatics prediction deleterious | 6 | | | |
| | | | | |
| Total number of INDELs | 13772 | 12761 | 11843 | 13281 |
| INDELs in coding region or splice site | 962 | 994 | 980 | 995 |
| Excluding non-framshift insert or deletion | 621 | 641 | 625 | 675 |
| MAF≤0.5% in 1000Genomes,ESP6500,ExAC | 44 | 52 | 50 | 57 |
| Shared variants in all patients | 6 | | | |
| Bioinformatics prediction deleterious | 0 | | | |
| | | | | |
| Candidate causative genes | 5 (6 distinct variants) | | | |
