# Supplementary material for: Identification of ANKDD1B variants in an ankylosing spondylitis pedigree and a sporadic patient
Source: BMC Med Genet. 2018 Jul 5;19:111. doi: 10.1186/s12881-018-0622-9 (PMC6034262; doi:10.1186/s12881-018-0622-9)
Supplement: Supplementary file 4 — Table S4. List of candidate genes. (PPTX 46 kb) [file 12881_2018_622_MOESM4_ESM.pptx]

## Slide 1
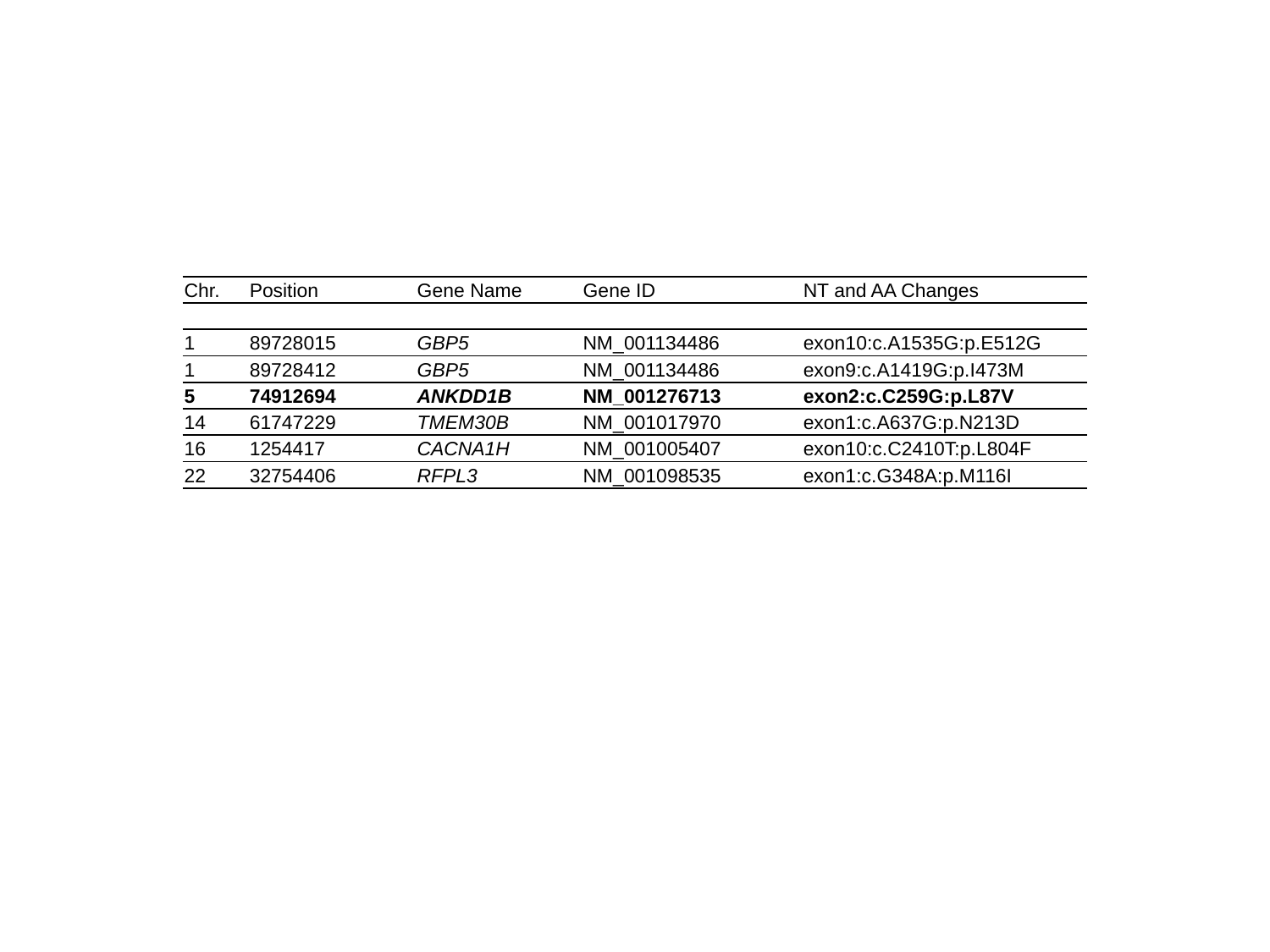

| | | | | |
| --- | --- | --- | --- | --- |
| Chr. | Position | Gene Name | Gene ID | NT and AA Changes |
| | | | | |
| 1 | 89728015 | GBP5 | NM\_001134486 | exon10:c.A1535G:p.E512G |
| 1 | 89728412 | GBP5 | NM\_001134486 | exon9:c.A1419G:p.I473M |
| 5 | 74912694 | ANKDD1B | NM\_001276713 | exon2:c.C259G:p.L87V |
| 14 | 61747229 | TMEM30B | NM\_001017970 | exon1:c.A637G:p.N213D |
| 16 | 1254417 | CACNA1H | NM\_001005407 | exon10:c.C2410T:p.L804F |
| 22 | 32754406 | RFPL3 | NM\_001098535 | exon1:c.G348A:p.M116I |
