# Supplementary material for: Identification of ANKDD1B variants in an ankylosing spondylitis pedigree and a sporadic patient
Source: BMC Med Genet. 2018 Jul 5;19:111. doi: 10.1186/s12881-018-0622-9 (PMC6034262; doi:10.1186/s12881-018-0622-9)
Supplement: Supplementary file 5 — Table S5. ANKDD1B PCR and sequencing primers. (PPTX 40 kb) [file 12881_2018_622_MOESM5_ESM.pptx]

## Slide 1
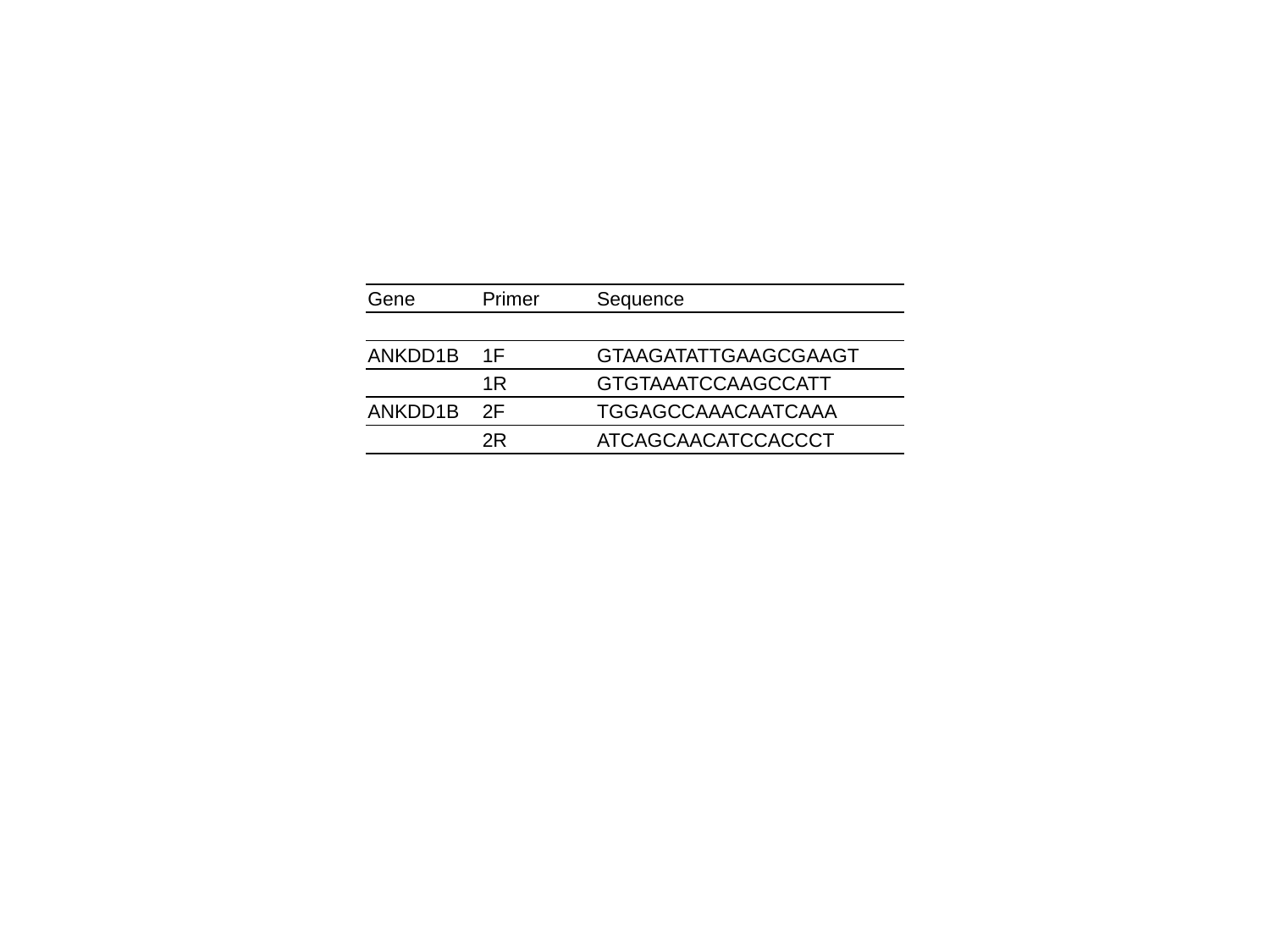

| | | |
| --- | --- | --- |
| Gene | Primer | Sequence |
| | | |
| ANKDD1B | 1F | GTAAGATATTGAAGCGAAGT |
| | 1R | GTGTAAATCCAAGCCATT |
| ANKDD1B | 2F | TGGAGCCAAACAATCAAA |
| | 2R | ATCAGCAACATCCACCCT |
