# Supplementary material for: Identification of ANKDD1B variants in an ankylosing spondylitis pedigree and a sporadic patient
Source: BMC Med Genet. 2018 Jul 5;19:111. doi: 10.1186/s12881-018-0622-9 (PMC6034262; doi:10.1186/s12881-018-0622-9)
Supplement: Supplementary file 6 — Table S6. PCR primers for haplotype genotyping. (PPTX 41 kb) [file 12881_2018_622_MOESM6_ESM.pptx]

## Slide 1
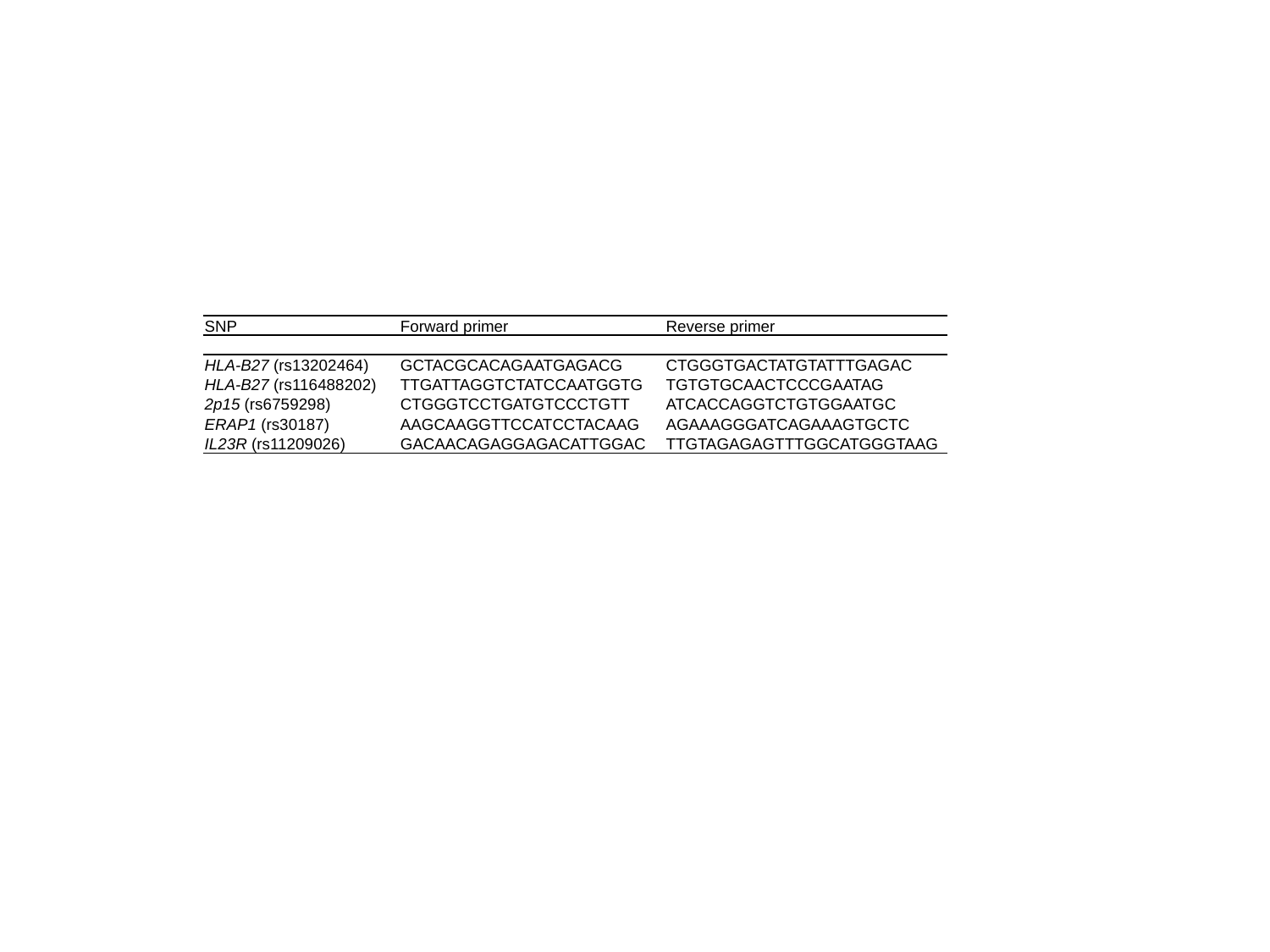

| SNP | Forward primer | Reverse primer |
| --- | --- | --- |
| | | |
| HLA-B27 (rs13202464) | GCTACGCACAGAATGAGACG | CTGGGTGACTATGTATTTGAGAC |
| HLA-B27 (rs116488202) | TTGATTAGGTCTATCCAATGGTG | TGTGTGCAACTCCCGAATAG |
| 2p15 (rs6759298) | CTGGGTCCTGATGTCCCTGTT | ATCACCAGGTCTGTGGAATGC |
| ERAP1 (rs30187) | AAGCAAGGTTCCATCCTACAAG | AGAAAGGGATCAGAAAGTGCTC |
| IL23R (rs11209026) | GACAACAGAGGAGACATTGGAC | TTGTAGAGAGTTTGGCATGGGTAAG |
