# Supplementary figures and images for: Identification of ANKDD1B variants in an ankylosing spondylitis pedigree and a sporadic patient
Source: BMC Med Genet. 2018 Jul 5;19:111. doi: 10.1186/s12881-018-0622-9 (PMC6034262; doi:10.1186/s12881-018-0622-9)

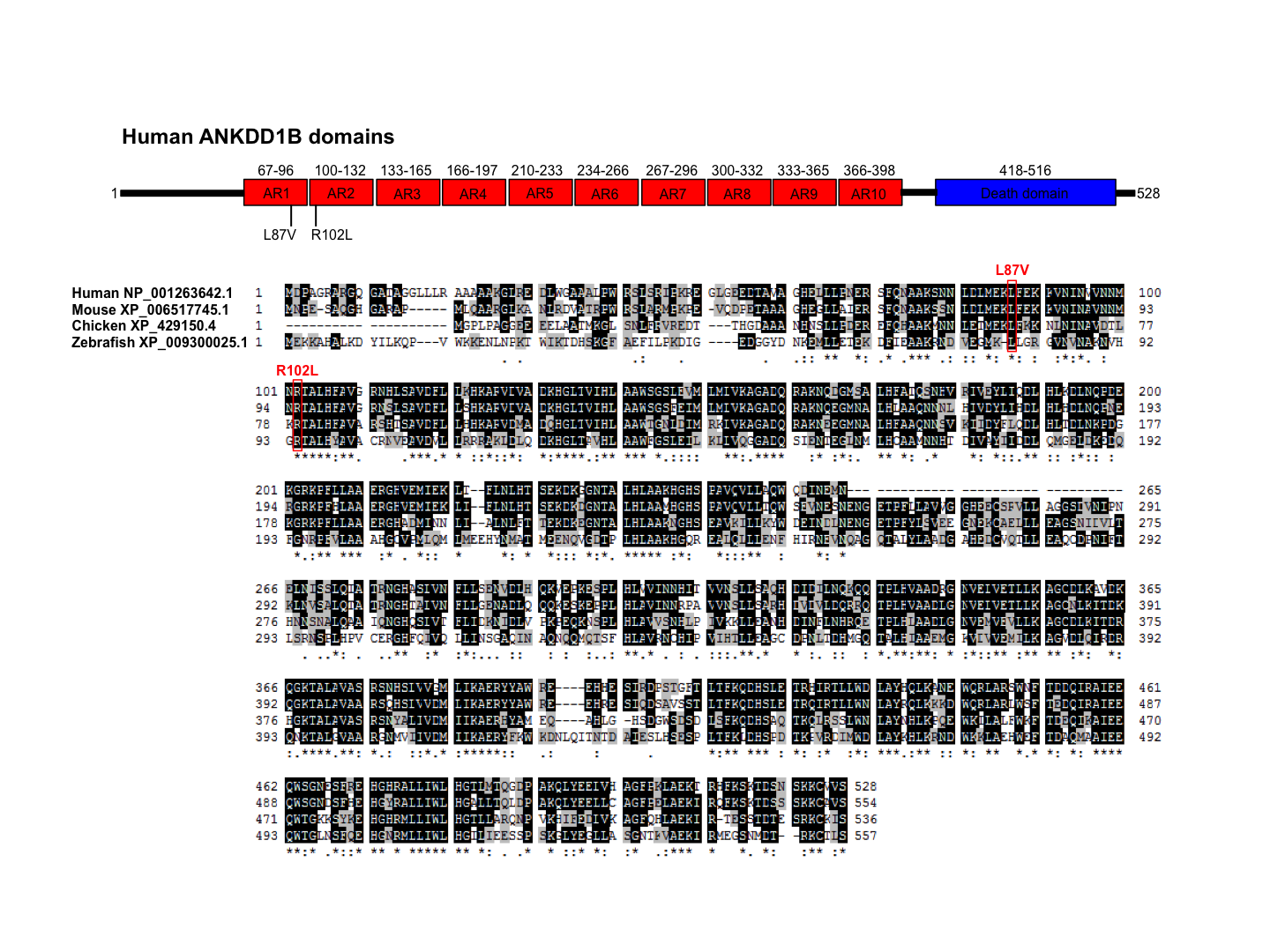

Supplement: Supplementary file 7 — Figure S1. ANKDD1B protein domains and sequence alignment. Upper panel: human ANKDD1B domain structure. AR: ankyrin repeat. Lower panel: ANKDD1B protein sequence alignment from zebrafish to human. The two variants identified in the AS9 pedigree and the sAS_P1 patient are indicated. (TIFF 6077 kb) [file 12881_2018_622_MOESM7_ESM.tiff]
